# Percutaneous transhepatic cholangioscopy- guided recanalization and reconstruction of Iatrogenic right hepatic duct occlusion with endoscopic assistance

**DOI:** 10.1055/a-2946-2091

**Published:** 2026-09-08

**Authors:** Tianhao Chen, Jingyi Zhang, Rui Chen, Jie Zhang, Wenjie Ma, Rongxing Zhou

**Affiliations:** 1 Division of Biliary SurgeryDepartment of General Surgery 34753 West China Hospital of Sichuan UniversityChengduSichuanChina; 2 Research Center for Biliary Diseases 34753 West China Hospital of Sichuan UniversityChengduSichuanChina; 3 Department of Ultrasound 34753 West China Hospital of Sichuan UniversityChengduSichuanChina

A 39-year-old woman presented with right upper quadrant pain and recurrent allergic skin eruptions 6 months after laparoscopic cholecystectomy. Magnetic resonance imaging showed a right hepatic duct stricture near the confluence with progressive intrahepatic ductal dilatation and right hepatic parenchymal changes ( Fig. 1 ), accompanied by elevated liver enzymes, suggesting iatrogenic bile duct injury (IBDI).

**Fig. 1:**
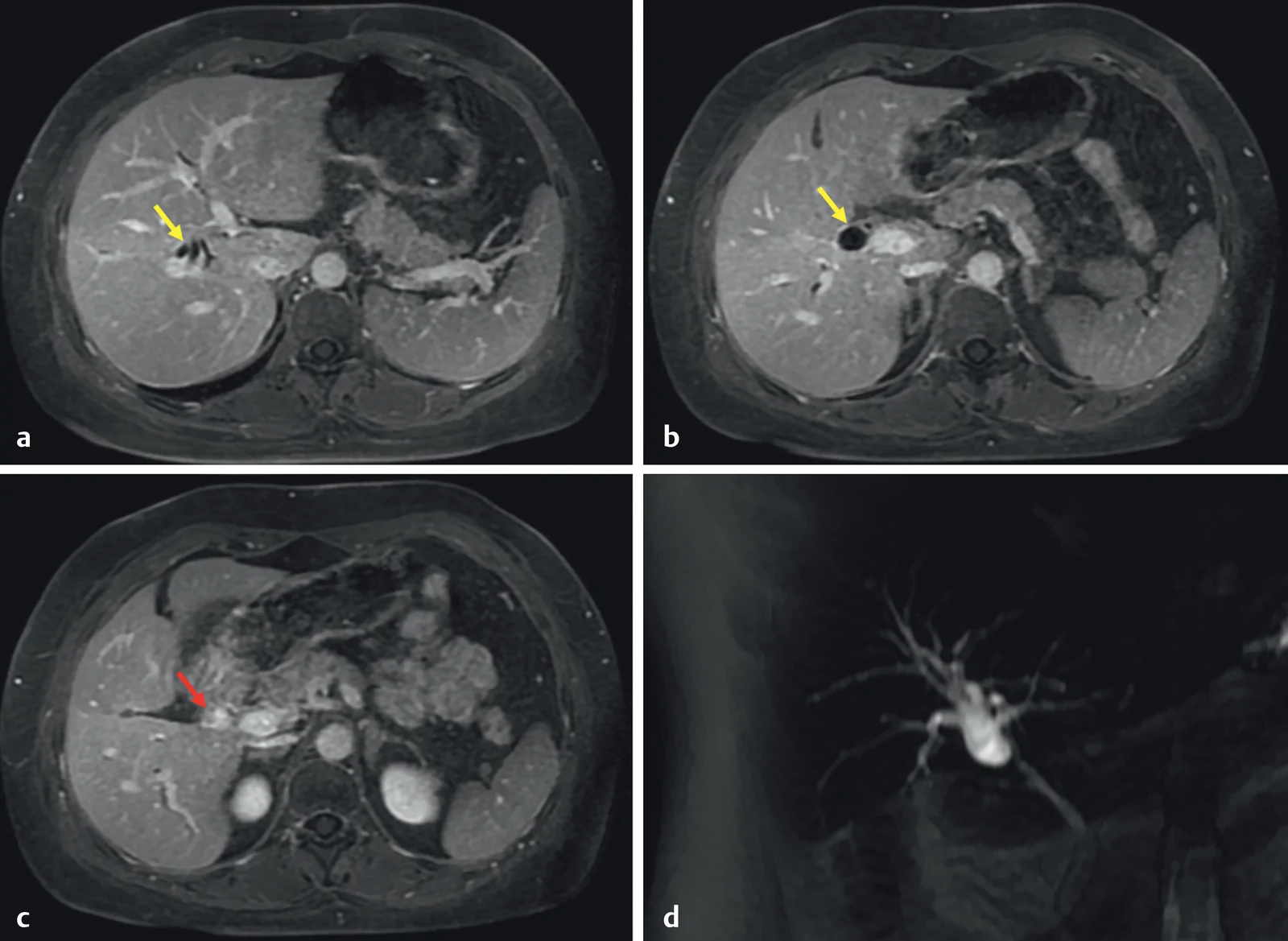
Preoperative MRI findings. ( **a** and **b** ) MRI showed dilation of the right hepatic duct (yellow arrows). ( **c** ) Stricture at the right hepatic duct confluence (red arrow). ( **d** ) MRCP demonstrated right-sided intrahepatic biliary dilation with stenosis at the right hepatic duct confluence. MRCP, Magnetic resonance cholangiopancreatography; MRI, magnetic resonance imaging.

Previous endoscopic retrograde cholangiopancreatography (ERCP) performed at another institution failed to traverse the stricture, and the patient was referred to our center for percutaneous transhepatic cholangioscopy (PTCS; Video 1 ). Cholangioscopy visualized the complete occlusion of the right hepatic duct with failed guidewire passage ( Fig. 2a ). Cholangiography confirmed no contrast passage into the common bile duct (CBD; Fig. 2b ). Rendezvous ERCP failed to traverse the occluded segment and reached only the left hepatic duct ( Fig. 2c ). A nasobiliary catheter was subsequently placed from the left hepatic duct into the CBD as a rendezvous landmark ( Fig. 2d ). Visualized re-puncture was performed across the occluded segment into the CBD using an 18-gauge needle ( Fig. 3a ) and the nasobiliary catheter was identified confirming successful access ( Fig. 3b ). Two weeks later repeat intervention confirmed complete recanalization and a covered metal stent was placed for biliary reconstruction, with cholangiography confirming patency ( Fig. 3c, d ).

**Video 1** PTCS-ERCP rendezvous recanalization of a completely occluded right hepatic duct.


**Fig. 2:**
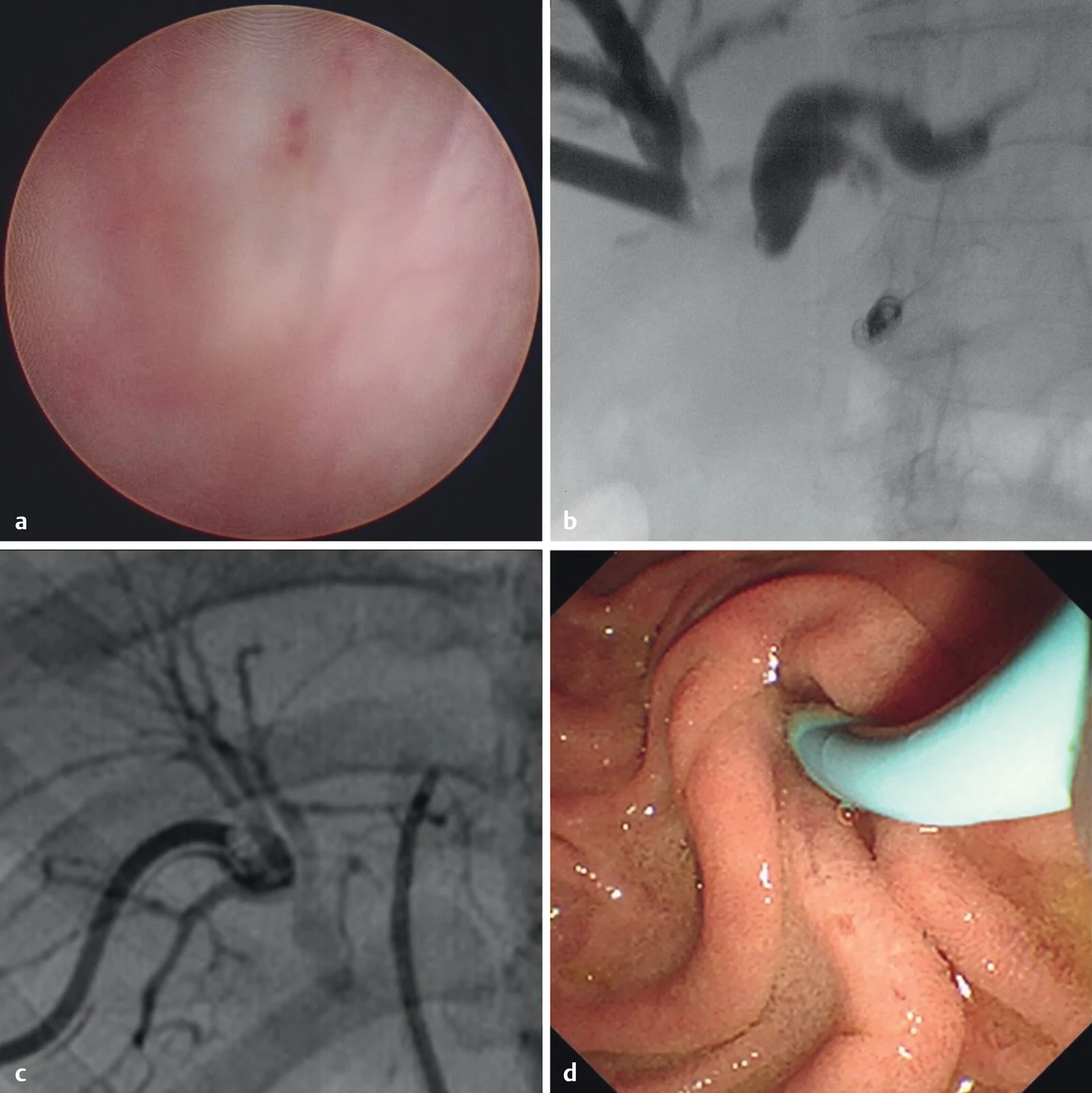
PTCS and ERCP findings during initial evaluation. ( **a** ) Direct cholangioscopy visualized the complete occlusion of the right hepatic duct. ( **b** ) Cholangiography confirmed the absence of contrast passage across the occluded segment. ( **c** ) Rendezvous ERCP failed to traverse the occluded segment and reached only the left hepatic duct. ( **d** ) A nasobiliary catheter was placed from the left hepatic duct into the common bile duct as a rendezvous marker.

**Fig. 3:**
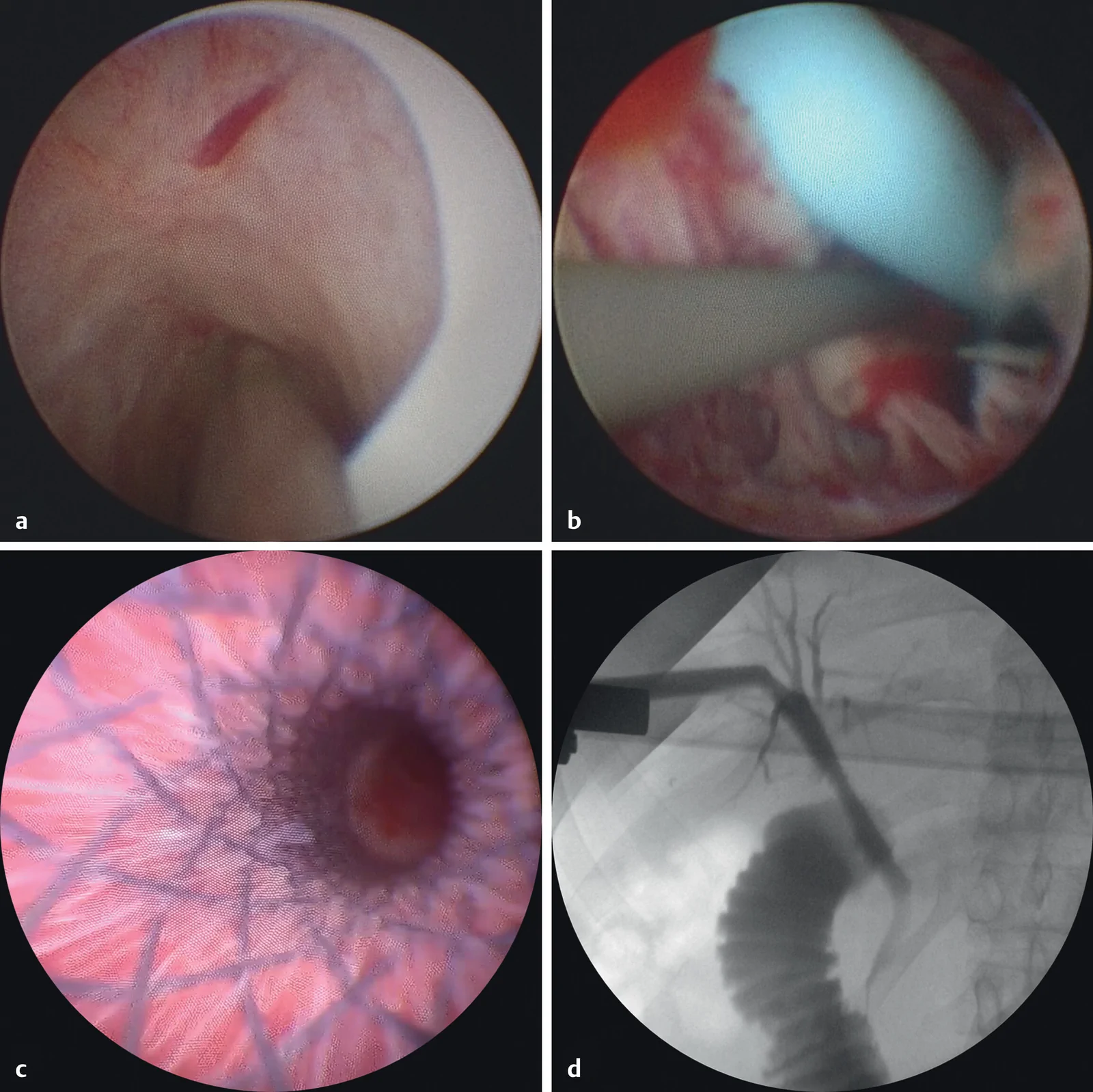
PTCS-ERCP rendezvous recanalization and biliary reconstruction. ( **a** ) Visualized re-puncture across the occluded segment was performed under combined cholangioscopic and ultrasound guidance. ( **b** ) The nasobiliary catheter was visualized after tract dilation, confirming successful recanalization. ( **c** ) A covered self-expanding metal stent was placed to maintain biliary patency. ( **d** ) Cholangiography confirmed the appropriate stent position and restored biliary patency.

During stent indwelling, liver function improved without recurrence of skin eruptions, while the patient continued oral ursodeoxycholic acid therapy. Repeat PTCS was performed for stent removal after 12 months. The stent remained patent without significant stone formation ( Fig. 4a, b, d ). After stent removal, no stricture was observed and the distal CBD was directly visualized ( Fig. 4c ).

**Fig. 4:**
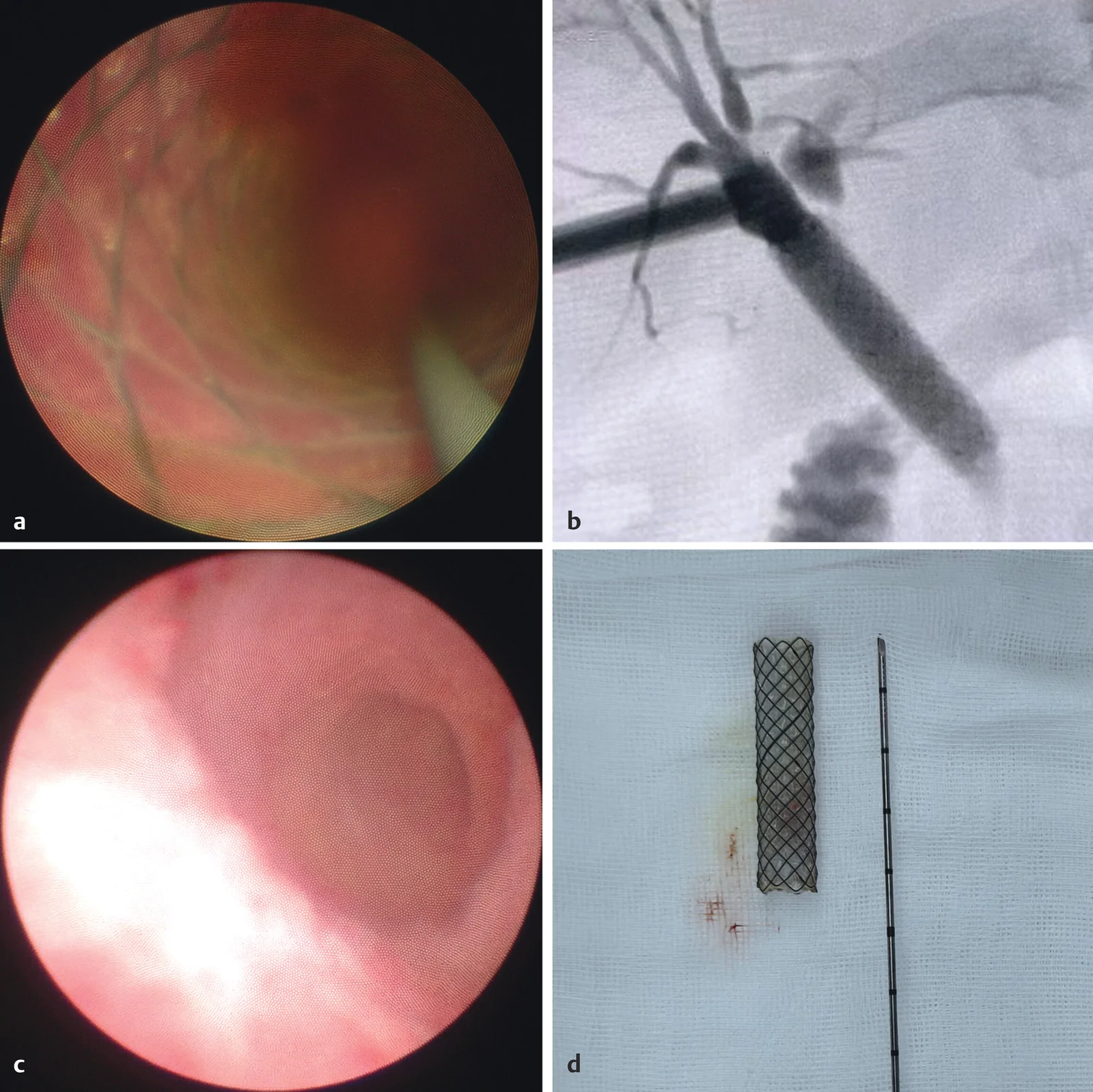
PTCS guided long-term stent management after biliary recanalization. ( **a** ) PTCS performed 12 months after stent placement showed a patent stent lumen. ( **b** ) Cholangiography confirmed sustained biliary patency. ( **c** ) After stent removal, no stricture was observed, with direct visualization of the distal common bile duct. ( **d** ) The removed covered self-expanding metal stent.

IBDI is a serious complication of laparoscopic cholecystectomy that may lead to severe long-term consequences. 1​ 2​ ​3​ PTCS is a valuable therapeutic option for biliary strictures, 4​ and this case illustrates the feasibility of combining PTCS with ERCP for recanalization of a completely occluded bile duct. 5​

Endoscopy_UCTN_Code_TTT_1AR_2AL

## Declaration of GenAI use

The authors confirm that no Generative AI was used in the preparation of this manuscript.
